# Phylogeography, Risk Factors and Genetic History of Hepatitis C Virus in Gabon, Central Africa

**DOI:** 10.1371/journal.pone.0042002

**Published:** 2012-08-01

**Authors:** Richard Njouom, Mélanie Caron, Guillaume Besson, Guy-Roger Ndong-Atome, Maria Makuwa, Régis Pouillot, Dieudonné Nkoghé, Eric Leroy, Mirdad Kazanji

**Affiliations:** 1 Centre International de Recherches Médicales de Franceville, Franceville, Gabon; 2 Centre Pasteur du Cameroun, Réseau International des Instituts Pasteur, Yaoundé, Cameroon; 3 Division of Infectious Diseases, University of Pittsburgh Medical Center, Pittsburgh, Pennsylvania, United States of America; 4 Ministère de la Santé, Libreville, Gabon; 5 Institut Pasteur de Bangui, Réseau International des Instituts Pasteur, Bangui, Central African Republic; University of Pretoria/NHLS TAD, South Africa

## Abstract

**Background:**

The epidemiological and molecular characteristics of hepatitis C virus (HCV) infection in the general population have been poorly investigated in Africa. The aim of this study was to determine the prevalence, genotype distribution and epidemic history of HCV in the Gabonese general population.

**Methods/Principal Findings:**

A total of 4042 sera collected from adults in 220 villages in all nine administrative areas of the country were screened for antibodies to HCV. HCV NS5B region sequencing was performed for molecular characterization and population genetic analyses. Of 4042 tested sera, 455 (11.2%) were positive. The seroprevalence of HCV varied significantly by administrative area, with the highest rate in Ogooué-Lolo province (20.4%) and the lowest in Ogooué-Maritine province (3.7%). History of parenteral injections, past hospital admission and age over 55 years were independent risk factors for HCV infection (p<0.0001). Phylogenetic analyses showed that 91.9% of the strains were genotype 4 (HCV-4), 5.7% genotype 1 and 2.2% genotype 2. HCV-4 strains were highly heterogeneous, with more than eight subtypes; subtype 4e predominated (57.3%). Coalescence analyses indicated that subtype 4e was the oldest, with an estimated most recent common ancestor of 1702 [95% CI, 1418–1884]. The epidemic profile indicated that it spread exponentially during the first part of the 20th century, probably by iatrogenic transmission.

**Conclusions/Significance:**

These results confirm the endemicity of HCV subtype 4e in Gabon and show that its spread is due to a cohort effect, with previous, possibly iatrogenic events. More extensive epidemiological studies are needed to better characterize the route of transmission and the dissemination of HCV in Gabon.

## Introduction

Hepatitis C virus (HCV) is a leading cause of chronic liver disease, cirrhosis, and hepatocellular carcinoma [1]. HCV infects about 170 million people worldwide (3% of the world’s population) and is recognized as a major public health problem [2]. Like Egypt [3], Central Africa is considered a high-prevalence region, antibodies against HCV being detected in more than 6% of the population [4]. The highest prevalences in these countries are typically observed in the oldest population groups [5]–[10].

HCV evolves very rapidly, resulting in high genetic diversity. It is classified into six genotypes, each subdivided into multiple subtypes [11]. Some subtypes are found only in particular regions, while others are distributed globally. Genotype 1a is most commonly detected in Europe and the USA, while genotype 1b is distributed worldwide with high prevalences in Europe and the USA as well as in Japan. Genotype 2 originated from West and Central Africa and is common in Europe, Japan, and North America. Genotype 3 is most common in India, Indonesia and South-East Asia. Genotype 4 appears to be prevalent in Central Africa and the Middle East, while genotype 5 is most frequently reported in South Africa and genotype 6 in Hong Kong and South-East Asia [12]. The time of divergence of HCV genotypes circulating in those regions has been estimated: the age of the most recent common ancestor (MRCA) of genotypes 1 and 4 in Central Africa and of genotype 2 in West Africa was estimated to be 500–600 years [13]–[16], whereas the age of the MRCA of genotype 6 in East Asia was dated to 1100–1350 years [12], supporting the idea of long-term endemic transmission of HCV in these regions. Whereas circulation of most HCV subtypes remains restricted to geographical regions with long-term endemic transmission of the virus, a few HCV subtypes (1a, 1b, and 3a) are highly prevalent ‘epidemic’ strains that have spread outside the endemic regions and are distributed globally [17]. It has been estimated that epidemic subtypes 1a, 1b, and 3a emerged only about 100–150 years ago and started to spread exponentially during the 20th century [18]–[24]. Recent emergence and expansion is fully consistent with the idea that worldwide dissemination of the epidemic HCV subtypes is mainly due to the emergence of new, efficient routes of viral transmission during the 20th century, such as blood transfusion, hemodialysis, injection drug use, and non-sterile medical injections [23], [25], [26].

In Gabon, the epidemiological picture is based on a few population-based surveys conducted since 1993. A seroprevalence of more than 6.5% was reported in Gabon [6], [27], and the available sequences indicated a predominance of genotype 4 [6], [28]. The only study on the genetic diversity of HCV NS5B sequences in Gabon showed that subtype 4e predominated in the 22 samples, and evolutionary analysis of the 4e sequences indicates a period of increased transmission during the early 20th century [6]. These population-based surveys have, however, been restricted to small towns and are not representative of the general population. As large-scale population-based studies are not available in Gabon, the results cannot be used to estimate the prevalence of HCV infection, the genotype distribution or the burden of the disease in the general population.

We present here the results of a population-based study in all nine provinces of Gabon. The purpose of the study was to determine the seroprevalence of HCV in a stratified sample (by age and geographical area) of the general rural population and to establish the distribution of viral subtypes. The epidemic history of HCV was deduced by analysis of independent sequences in the NS5B region with new coalescence techniques [22].

## Methods

### Study Area and Population

Gabon is located in central Africa, transversed by the equator; nearly 80% is covered by rain forest. The country has a surface area of 267 667 km^2^ with about 1.5 million inhabitants (5.6 inhabitants/km^2^), 73% of whom live in urban areas. Administratively, Gabon is divided into nine provinces with 2048 villages located mainly along roads and rivers; few have more than 300 inhabitants. The main activities are subsistence farming, hunting, gathering and fishing. This study was conducted on blood samples collected between June 2005 and September 2008, during a project on Ebola virus in Gabon [29]. Briefly, the survey covered 220 randomly selected villages in the nine administrative regions of Gabon (range, 10–41 villages per province), most of which were rural with fewer than 300 inhabitants. The multidisciplinary team comprised a doctor from the Gabonese Ministry of Health, a nurse, an epidemiologist, a virologist and laboratory technicians, who made nine 1-month field missions to the study villages. All healthy volunteers over 15 years old who had been residing in the village for more than 1 year were eligible for the study.

The study protocol was reviewed and approved by the Gabonese Ministry of Health (Research authorization No. 00093/MSP/SG/SGAQM). The Health Director and the Governor of each province received written information, as did the traditional chief of each village. The planned studies were described orally to all participants, and individual written consent was obtained for blood sampling; parents’ written consent was obtained for participating children and minors. People who gave informed consent were interviewed on a structured epidemiological questionnaire covering history of blood transfusion, jaundice, mass treatment and mass vaccination, sharing toiletry items with other family members, suspected nosocomial transmission (surgical and dental procedures, hospitalizations) and suspected sporadic transmission (ritual scarring, ritual circumcision for men, ritual ear-piercing for women). A free medical examination and basic medicines were provided to all participants and non-participants. Blood smears for malaria diagnosis and field blood typing were also proposed.

Blood samples were usually collected in the village health care centers into 7-ml Vacutainer tubes containing EDTA (VWR International, France). The tubes were transported to the field laboratory for centrifugation (10 min, 2000 × *g*) daily. Plasma and buffy coat were stored in aliquots at –20°C until the end of the field mission and then transferred on dry ice to the Centre International de Recherches Médicales de Franceville (CIRMF) and kept at –80°C until analysis. Each plasma sample was separated upon collection into two aliquots: one for serological testing and the other for molecular characterization.

### Serological Test

The presence of antibodies to HCV was checked with a third-generation enzyme immunoassay (Monolisa anti-HCV plus version 2, Biorad, Marnes-La-Coquette, France). The reactivity of samples was determined as described previously [30]. Briefly, a ratio was calculated for each sample by dividing its optical density by the cut-off value. A sample was scored as positive if the ratio was ≥6, whereas all samples with a ratio <6 were scored as negative.

### HCV Genotyping and Subtyping

HCV genotyping and subtyping were performed by amplification, sequencing and phylogenetic analysis of a 382-nt fragment of the NS5B gene. Briefly, viral RNA was extracted from 140 µl of plasma from HCV-positive individuals with a QIAamp Viral RNA Mini Kit according to the manufacturer's protocol (Qiagen, Courtaboeuf, France). The portion of HCV NS5B gene from extracted RNA was further amplified and sequenced as described previously [31]. Briefly, RNA was subjected to hemi-nested polymerase chain reaction (PCR) amplification, with Pr3 (5′-TATGAYACCCGCTGYTTTGCTC-3′) and Pr4 (5′-GCNGARTAYCTVGTCATAGCCTC-3′) as primers in the first step and Pr3 and Pr5 (5′-GCTAGTCATAGCCTCCGT-3′) in the second step. Combined reverse transcriptase (RT)-PCR was carried out on 10 µl of extracted RNA (reaction mixture volume, 50 µl) with the one-step RT-PCR kit (Superscript III) and 200 nM of each primer (Pr3, Pr4 and Pr5). The first step of RT-PCR with Pr3 and Pr4 was carried out at 50°C for 30 min, then five cycles at 93°C for 30 s, 60°C for 45 s and 72°C for 1 min, followed immediately by 35 cycles at 93°C for 30 s, 60°C with a drop of −0.3°C between each cycle and elongation at 72°C for 1 min. The final elongation was at 72°C for 5 min. The second amplification step with Pr3 and Pr5 was carried out on 2 µl of the first PCR products at 95°C for 5 min, then 35 cycles at 95°C for 30 s, 55°C for 30 s, 72°C for 30 s and a final elongation at 72°C for 10 min. The amplified NS5B 382-bp product was analyzed by electrophoresis with a 2% agarose gel and ethidium bromide staining. PCR products were purified on columns (Quick-Spin™ Qiagen, Hilden, Germany) and sequenced. PCR primers (Pr3 and Pr5) were used for sequencing both DNA strands. Cycle sequencing was undertaken by the fluorescent dye terminator technique (Big Dye Terminator Cycle sequencing; Applied Biosystems, Courtaboeuf, France) with Ampli Taq™ DNA polymerase according to the manufacturer's instructions. Electrophoresis and data collection were done on an Applied Biosystems ABI 3100 Genetic Analyzer. Sequence chromatograph files were analyzed with sequence analysis™ and sequence navigator™ software.

For phylogenetic analysis, NS5B nucleotide sequences were aligned initially by CLUSTAL_X 1.81 [32] and subsequently adjusted by hand. The sequences were compared with reference sequences from the European HCV database (http://euhcvdb.ibcp.fr/euHCVdb/) and the Los Alamos database (http://hcv.lanl.gov/). Phylogenetic trees were estimated and assessed by the bootstrapping and neighbor-joining methods under the Kimura two-parameter substitution model, as implemented in MEGA version 5.0 [33]. Bootstrapping was performed with 1000 replicates.

In order to confirm the results obtained by NS5B genotyping and to identify probable recombinant HCV isolates, a 360-nucleotide fragment of the core gene was amplified as described previously [34] from a set of randomly selected samples. Briefly, RNA was subjected to a nested PCR amplification with CoreOS (5′-ACTGCCTGATAGGGTGCTTGCGAG-3′) and CoreOAS (5′-ATGTACCCCATGAGGTCGGC-3′) as the primers in the first step and CoreIS (5′-AGGTCTCGTAGACCGTGCATCATG-3′) and CoreIAS (5′-CAYGTRAGGGTATCGATGAC-3′) in the second step. cDNA synthesis was first was carried out on 10 µl of extracted RNA (reaction mixture volume, 20 µl) with the RT kit (AMV-RT, Promega) and a random hexamer. The first step of PCR with CoreOS and CoreOAS and the second PCR with CoreIS and CoreIAS were carried out at 94°C for 3 min, then two cycles at 95°C for 30 s, 60°C for 30 s, and 72°C for 30 s, followed immediately by two of the same cycles with a drop of −1°C at each hybridation cycle until 51°C and then 20 cycles of 95°C for 30 s and 50°C for 30 s, with a final elongation at 72°C for 7 min. The amplified core 360-bp product was analyzed by electrophoresis on a 2% agarose gel with ethidium bromide staining. The PCR products were purified and sequenced as described above. The PCR primers CoreIS and CoreIAS were used for sequencing both DNA strands.

### Coalescent Analysis

The epidemic history of HCV subtypes 4e, 4f, 4c, 4t, and 4k, the prevalences of which represent at least 5% of circulating HCV isolates in Gabon, was investigated with a coalescent-based strategy. Briefly, HCV demographic history was inferred by Bayesian Monte Carlo Markov Chain (MCMC) analysis in BEAST 1.4 software (http://.beast.bio.ed.ac.uk), as described elsewhere [14]. We used an informative prior normal distribution, with a mean of 5.0 × 10^–4^ and a standard deviation of 7.14 × 10^–5^. This distribution represents a best estimate of HCV NS5B evolutionary rates, as obtained from two independent prior analyses [22], [24]. As recommended, a relaxed molecular clock approach (uncorrelated lognormal model) was used, thereby taking into account the variation in evolutionary rate among lineages [35]. BEAST output files were analyzed with TRACER 1.3 (http://tree.bio.ed.ac.uk/software/tracer/).

### Statistical Analysis

Statistical analysis was performed with Epi-Info (version 6.04dfr, ENSP-Epiconcept-InUS, 2001). The overall prevalence was checked by the chi-squared test with Yates correction. *p* values <0.05 were considered statistically significant. Data were then analyzed by logistic regression. The continuous variable age was categorized and entered into the model. The strength of the association between age and HCV infection was estimated in the multivariate analyses as odds ratios (ORs) with 95% confidence intervals (CIs).

### Nucleotide Sequence Accession Numbers

The nucleotide sequences obtained from the core and NS5B gene of HCV were assigned GenBank accession numbers JN642718–JN642776 (59 sequences) and JN642777–JN642987 (211 sequences), respectively.

## Results

### Study Population

**Figure 1:**
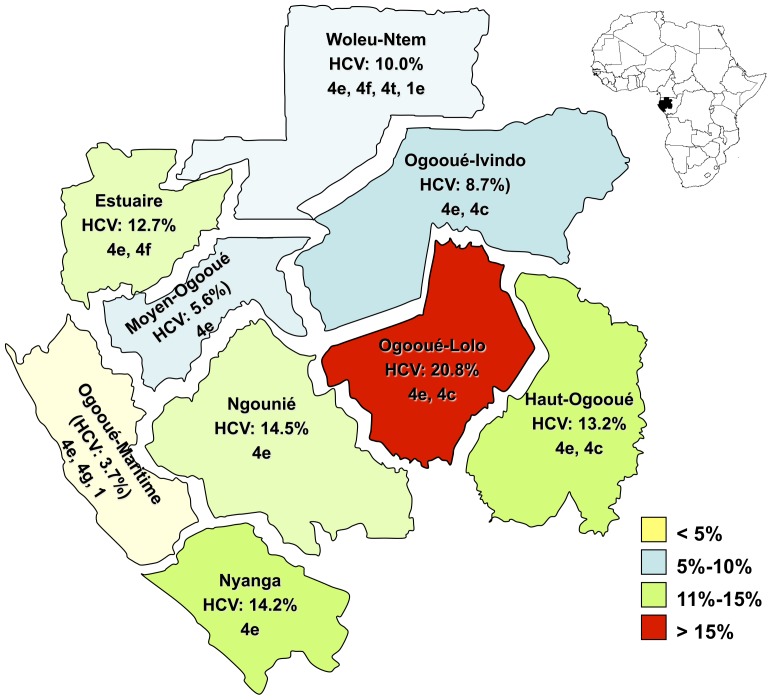
Map of Gabon with administrative regions, prevalence of antibodies to HCV and predominant subtypes.

We enrolled 4042 people in 220 randomly selected villages covering all nine administrative areas of the country (Figure 1). Blood samples and sociodemographic data were collected from all participants. The mean age ± SD of the study population was 47±14.3 years (range, 15–90 years) (Table 1), and the sex distribution was 2180 (53.9%) females and 1860 (46.1%) males. There was no significant difference in mean age by province.

**Table 1:** Distribution and mean age of study population in the nine administrative areas of Gabon.

Province	Number of participants	Mean age ± SD (years)
Estuaire	314	46±12.7
Haut Ogooué	363	45±13.3
Moyen Ogooué	676	45±14.7
Ngounié	303	48±14.4
Nyanga	422	46±12.0
Ogooué Ivindo	457	46±14.6
Ogooué Lolo	423	52±13.9
Ogooué Maritime	187	50±18.7
Woleu Ntem	897	47±14.6
Total	4042	47±14.3

### HCV Seroprevalence, Regional and Age Distribution and Risk Factors for Infection

The seroprevalence of HCV in the study population was 11.2% (95% CI, 10.3–12.3) (Table 2), with no significant difference between females (11.0%; 95% CI, 9.7–12.4) and males (11.6%; 95% CI, 10.2–13.2).

**Table 2:** Prevalence of hepatitis C virus infection according to age group and administrative area in Gabon.

Number of people with antibodies to HCV/number of people tested										
(%; 95% confidence interval)										
Age(years)	Estuaire	Haut Ogooué	Moyen Ogooué	Ngounié	Nyanga	O. Ivindo	O. Lolo	O. Maritime	Woleu Ntem	Total
≤25	1/26	0/38	0/75	1/29	3/32	0/58	0/17	0/17	1/79	6/371
	(3.8;0.1–19.6)	(0.0;0.0–9.3)	(0.0;0.0–4.8)	(3.4;0.09–17.8)	(9.4;2.0–25.0)	(0.0;0.0–9.3)	(0.0;0.0–19.5)	(0.0;0.0–19.5)	(1.3;0.0–6.9)	(1.6;0.6–3.5)
26–35	2/45	0/56	2/132	2/34	2/65	0/67	2/50	0/41	0/127	10/617
	(4.4;0.5–15.1)	(0.0;0.0–6.3)	(1.5;0.2–5.4)	(5.9;0.7–19.7)	(3.1;0.4–10.7)	(0.0;0.0–5.4)	(4.0;0.5–13.7)	(0.0;0.0–8.6)	(0.0;0.0–2.9)	(1.6;0.8–3.0)
36–45	3/74	3/76	3/127	4/51	6/79	3/86	9/77	0/23	10/166	42/759
	(4.1;0.8–11.4)	(3.9;0.8–11.1)	(2.4;0.5–6.7)	(7.8;2.2–18.9)	(7.6;2.8–15.8)	(3.5;0.7–9.9)	(11.7;5.5–21.0)	(0.0;0.0–14.8)	(6.0;2.9–10.8)	(5.5;4.0–7.4)
46–55	9/87	16/80	10/136	13/67	18/131	8/113	17/70	2/30	16/184	111/898
	(10.3;4.8–18.7)	(20.0;11.9–30.4)	(7.4;3.6–13.1)	(19.4;10.8–30.9)	(13.7;8.4–20.8)	(7.1;3.1–13.5)	(24.3;14.8–36.0)	(6.7;0.8–22.1)	(8.7;5.1–13.7)	(12.4;10.3–14.7)
>55	25/82	28/113	23/206	22/122	31/115	29/133	60/209	5/78	63/341	286/1397
	(30.5;20.8–41.6)	(24.8;17.1–33.8)	(11.2;7.2–16.3)	(18.0;11.7–26.0)	(27.0;19.1–36.0)	(21.8;15.1–29.8)	(28.7;22.7–35.4)	(6.4;2.1–14.3)	(18.5;14.5–23.0)	(20.5;18.4–22.7)
Total	40/314	48/363	38/676	44/303	60/422	40/457	88/423	7/187	90/897	455/4042
	(12.7;9.3–16.9)	(13.2;9.9–17.1)	(5.6;4.0–7.6)	(14.5;10.8–19.0)	(14.2;11.0–17.9)	(8.7;6.3–11.7)	(20.8;17.0–25.0)	(3.7;1.5–7.6)	(10.0;8.1–12.2)	(11.2;10.3–12.3)

O, Ogooué.

The seroprevalence of HCV varied significantly according to administrative area (Figure 1, Table 2). The seroprevalence in Ogooué-Lolo province (20.8%; 95% CI, 17.0–25.0) was significantly higher than that in the other provinces (*p*<0.001), while that in Ogooué-Maritime province (3.7%; 95% CI, 1.5–7.6) was significantly lower (*p*<0.001). Four patterns of seroprevalence were found (Figure 1): <5% (Ogooué-Maritime), 5–10% (Moyen-Ogooué, Ogooué-Ivindo and Woleu-Ntem), 11–15% (Estuaire, Nyanga, Ngounié, and Haut-Ogooué), and >15% (Ogooué-Lolo).

As shown in Table 2, the seroprevalence increased with age, from 1.6% in the <25-year age group to 12.4% at 46–55 years and 20.5% at >55 years (chi squared test for trend, *p*<0.001). This trend was similar in all geographical areas, with significant increases with increasing age, the highest prevalence being in the >55-year age group.

Univariate analysis of risk factors for HCV infection among seropositive and seronegative people showed that the independent predictors of positivity for HCV antibodies were a history of parenteral injections (*p*<0.0001; OR, 1.86; 95% CI, 1.52–2.28), past hospital admission (*p*<0.0001; OR, 1.42; 95% CI, 1.15–1.75) and age >55 years (*p*<0.0001; OR, 3.77; 95% CI, 3.08–4.62) (Table 3). The same analyses were done separately for each administrative area, with similar results (data not shown, available upon request).

**Table 3:** Univariate analysis of risk factors for HCV infection among HCV-positive and HCV-negative individuals.

Risk factor	HCV positive, N (%)	HCV negative, N (%)	OR [95% CI]*	*p*
Gender				
Male	216 (11.6)	1644 (88.4)	1.00†	>0.05
Female	239 (11.0)	1941 (89.0)	0.94 [0.77; 1.14]	
Age (years)				
≤55	169 (6.4)	2476 (93.6)	1.00†	**<0.001**
>55	286 (20.5)	1111 (79.5)	3.77 [3.08; 4.62]	
Blood or blood product transfusion				
No	404 (11.4)	3138 (88.6)	1.00†	>0.05
Yes	41 (10.5)	351 (89.5)	0.91 [0.65; 1.28]	
Past hospital admission				
No	144 (9.3)	1402 (90.7)	1.00†	**<0.001**
Yes	305 (12.7)	2091 (87.3)	1.42 [1.15; 1.75]	
History of parenteral injections				
No	270 (9.5)	2577 (90.5)	1.00†	**<0.001**
Yes	180 (16.3)	923 (83.7)	1.86 [1.52; 2.28]	
Jaundice				
No	66 (7.8)	777 (92.2)	1.00†	>0.05
Yes	7 (11.5)	54 (88.5)	1.53 [0.67; 3.50]	
Traditional scarification				
No	230 (12.0)	1693 (88.0)	1.00†	>0.05
Yes	220 (10.9)	1805 (89.1)	0.90 [0.74; 1.10]	

* OR, odds ratio; CI, confidential interval; †, reference.

### HCV Genotype and Subtype Distribution

**Figure 2:**
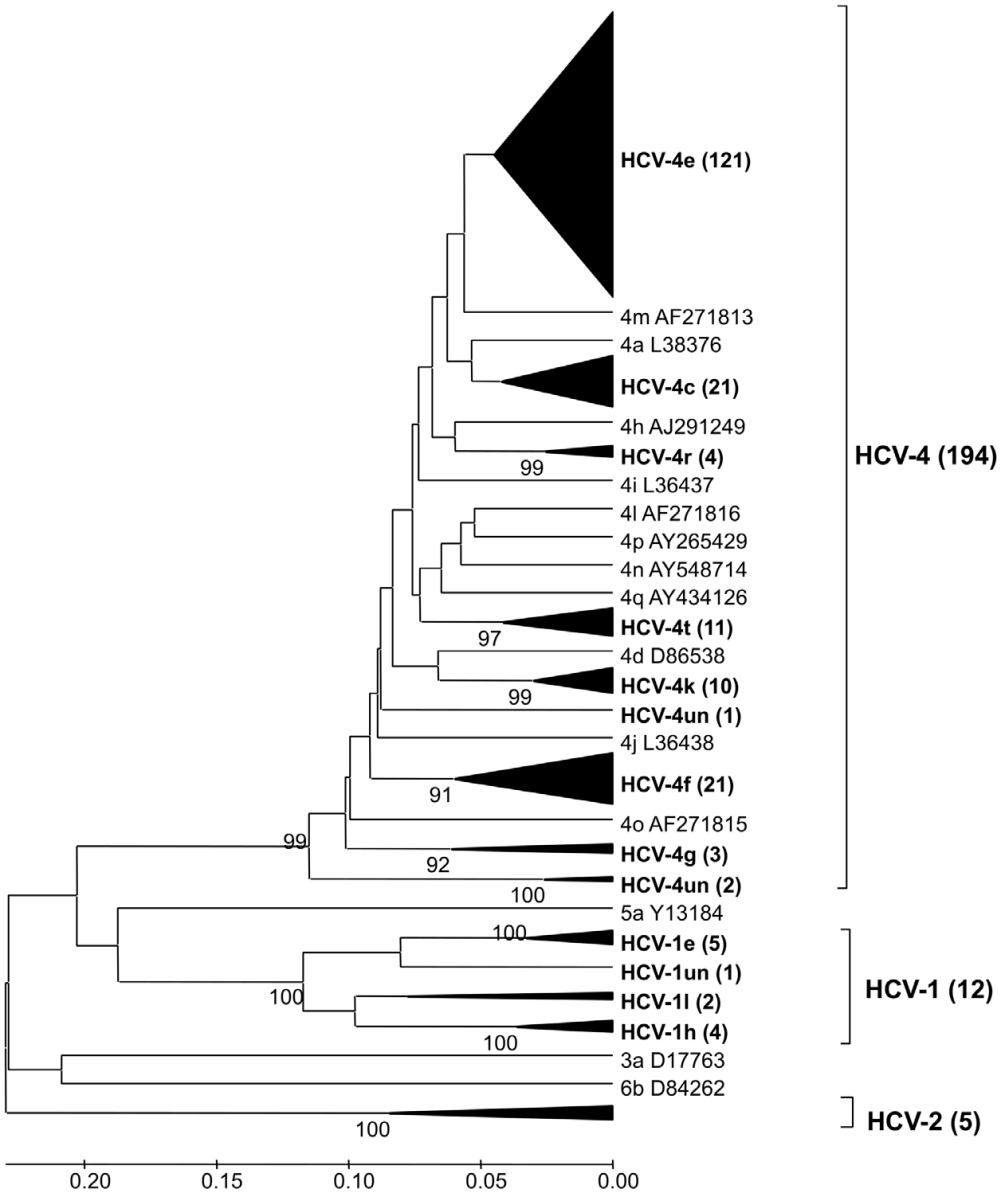
Phylogram depicting the phylogenetic relations between partial (380-bp) HCV NS5B sequences obtained in this study and representative sequences of HCV genotypes fromGenBank. The sequences from this study are shown in bold and grouped in their corresponding clusters. Percentage bootstrap values (>70%) are shown at the respective nodes. All 211 sequences were submitted to GenBank with the accession numbers JN642777–JN642987.

HCV RNA in the NS5B region could be amplified, sequenced and analysed phylogenetically from 211 of the 455 HCV-seropositive participants (46.4%). Figure 2 shows the estimated phylogeny of these and previously published HCV NS5B sequences. Of the 211 HCV samples, 194 (91.9%) were genotype 4 (HCV-4), 12 (5.7%) were genotype 1, and five (2.2%) were genotype 2. Within genotype 4, more than half the sequences (121, 57.3%) were in one cluster, i.e. reference 4e subtype sequences; 21 (9.9%) were associated with 4c sequences, 21 (9.9%) with 4f, 11 (5.2%) with 4t, 10 (4.7%) with 4k, 4 (1.9%) with 4r, and 3 (1.4%) with 4g. The remaining three (1.3%) sequences did not cluster with current subtype HCV-4 sequences. Within genotype 1, a cluster of five sequences (41.7%) corresponded to subtype 1e and four (33.3%) to 1h. Two sequences were subtyped as 1l, and one was an unclassified HCV-1 subtype. The five HCV genotype 2 sequences formed a monophyletic cluster and were unclassified.

The genotype/subtype distribution of 211 HCV-RNA positive samples according to geographical area is shown in Table 4. Except in Woleu-Ntem province, subtype 4e was the most prevalent. In some provinces, particularly those located in the centre of the country, subtype 4e was found in more than 50% of the typed samples. In the provinces in which subtype 4e represented less than 50% of subtypes, a high level of co-circulation (>20%) with other subtypes of genotype 4 was observed. These provinces were principally those that border other central African countries, i.e Woleu-Ntem and Estuaire bordering Cameroon with subtype 4f and Haut-Ogooué bordering the Republic of Congo with subtype 4c (Figure 1 and Table 4).

**Table 4:** Distribution of hepatitis C virus genotypes and subtypes according to administrative area in Gabon.

	Number of positive samples (% prevalence)									
HCVgénotypeor subtype	Estuaire(N = 28)	Haut Ogooué(N = 27)	Moyen Ogooué(N = 29)	Ngounié(N = 25)	Nyanga(N = 24)	Ogooué Ivindo(N = 25)	OgoouéLolo(N = 24)	WoleuNtem(N = 24)	Ogooué Maritime(N = 5)	Total(N = 211)
4e	**11 (39.3)**	**13 (48.1)**	**17 (56.6)**	**22 (88.0)**	**19 (79.2)**	**14 (56.0)**	**16 (66.7)**	**6 (25.0)**	**3 (60.0)**	**121 (57.3)**
4c	1 (3.6)	**9 (33.3)**	1 (3.4)	0	1 (4.2)	**4 (16.7)**	**5 (20.8)**	0	0	21 (9.9)
4f	**8 (28.6)**	0	2 (6.9)	1 (4.0)	0	2 (8.0)	1 (4.2)	**7 (29.2)**	0	21 (9.9)
4t	2 (7.1)	0	2 (6.9)	0	0	1 (4.0)	1 (4.2)	**5 (20.8)**	0	11 (5.2))
4k	3 (10.7)	0	3 (10.3)	2 (8.0)	2 (8.3)	0	0	0	0	10 (4.7)
4r	1 (3.6)	2 (7.4)	0	0	0	0	1 (4.2)	0	0	4 (1.9)
4g	0	0	0	0	2 (8.3)	0	0	0	**1 (20.0)**	3 (1.4)
4un	0	2 (7.4)	0	0	0	1 (4.0)	0	0	0	3 (1.4)
1	2 (7.1)	0	2 (6.9)	0	0	1 (4.0)	0	**6 (25.0)**	**1 (20.0)**	12 (5.7)
2	0	1 (3.7)	2 (6.9)	0	0	2 (8.0)	0	0	0	5 (2.4)

N, number of samples tested.

To validate the genotype/subtype assignment based on analysis of the NS5B region, sequence analysis was performed on the core region for 59 randomly selected isolates. The phylogenetic tree (data not shown) confirmed the subtype assignment for all isolates.

### Epidemic History of the Commonest Subtypes of HCV Circulating in Gabon

In order to investigate the origin and spread of HCV-4 in this population more carefully, subtypes with at least 10 NS5B sequences (4e, 4f, 4c, 4t, and 4k) were selected, and the divergence date and epidemic history were estimated with a Bayesian coalescent approach.

Table 5 shows the date of the MRCA for the five most prevalent Gabonese HCV-4 subtypes. Subtype 4e strain was the oldest, with an estimated MRCA date of 1702 (95% CI, 1418–1884); the dates of the 4f, 4c and 4t MRCAs were in the same range and were estimated to be 1888 (95% CI, 1855–1915), 1881 (95% CI, 1813–1930), and 1875 (95% CI, 1820–1918), respectively, while the 4k MRCA appeared to be more recent (1944; 95% CI, 1922–1962).

**Table 5:** Descriptive statistics from the empirical posterior distribution of the date of the most recent common ancestors (MRCAs) of Gabonese HCV NS5B partial sequences according to HCV-4 subtype.

Subtype	n	Median date (years)	95% CI date (years)
4e	121	1702	1418–1884
4f	21	1888	1855–1915
4c	21	1881	1813–1930
4t	11	1875	1820–1918
4k	10	1944	1922–1962

These distributions were obtained by Bayesian inferences with BEAST software, with a normal posterior distribution of the mean mutation rate at a mean of 5×10^4^ and a standard deviation of 7.14×10^5^. 95% credible confidence intervals are delimited by the 0.025th and 0.975th quantiles.

**Figure 3:**
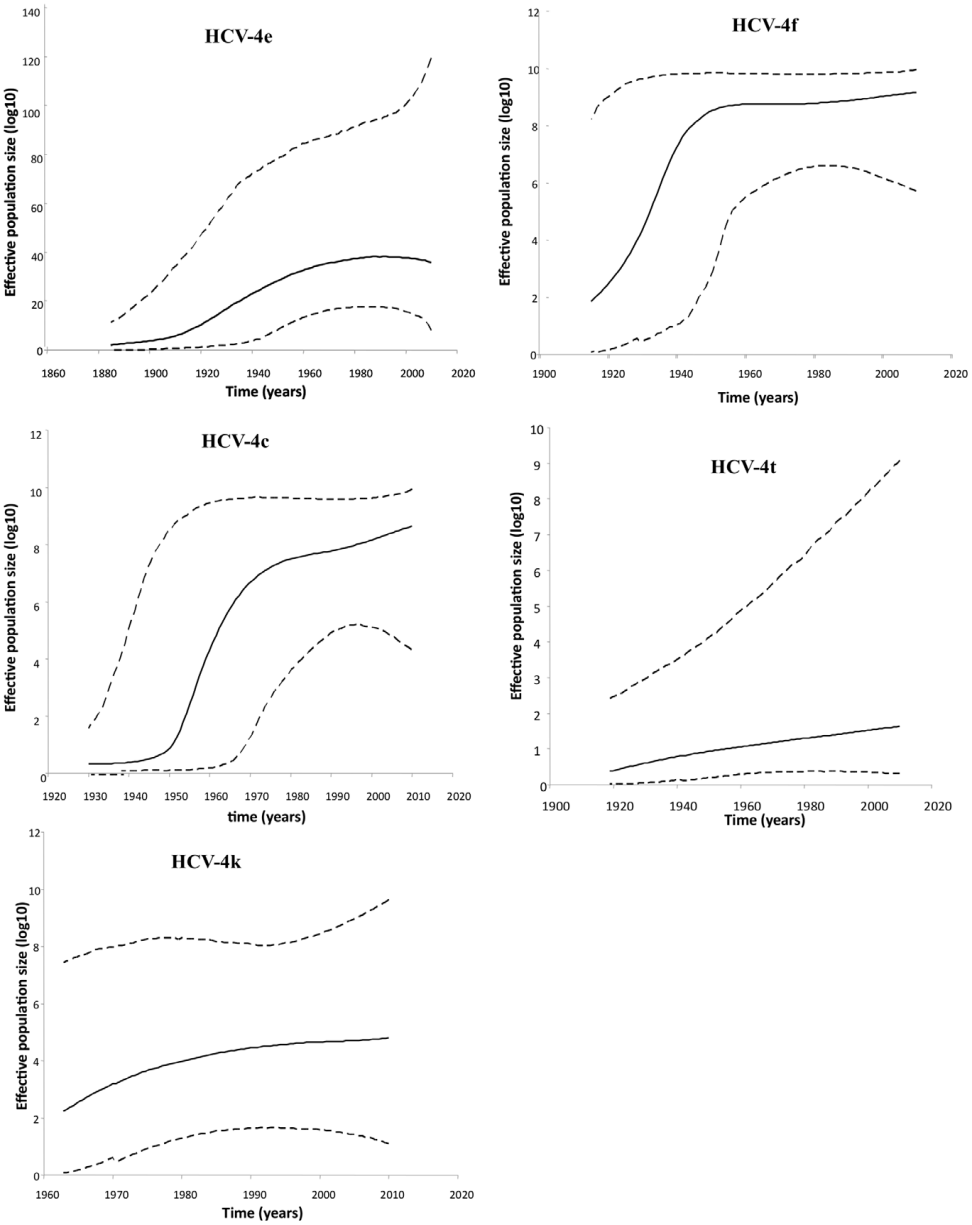
Bayesian skyline plots estimated for HCV subtypes 4e, 4f, 4c, 4t, and 4k in Gabon. The middle line is the median estimate of effective population size, and the envelope shows the 95% highest posterior density interval of this estimate.

The Bayesian skyline plot (Figure 3) depicts the estimated change in the effective number of infected individuals over time, from the HCV-4 MRCA to the year of sampling. Two patterns of epidemic history were observed. The first was that of HCV 4e, 4f, and 4c, the epidemic history of which was characterized by three phases of population growth: an initial period of relatively constant population size, a period of exponential growth during the first part of the 20th century and, finally, slower exponential growth. The second pattern was that of HCV 4t and 4k, which was characterized by one phase of population growth, including a period of exponential growth during the first part of the 20th century. The epidemic history of 4c precedes that of 4e and 4f by several decades. While the 4c population increased exponentially between 1950 and 1980, the 4e and 4f populations experienced exponential growth between 1920 and 1960. In contrast, the 4t and 4k populations did not reach growth equilibrium during this period.

## Discussion

We report here the largest HCV seroepidemiological study performed in Gabon so far, with 4042 blood samples from all nine provinces of the country. Overall, the seroprevalence of HCV was 11.2%, thus confirming a high prevalence of HCV infection in Gabon. Its heterogeneity (ranging from 3.7% to 20.8%) depended on the area studied. Studies in other countries have also reported significant intra-country variation in HCV prevalence [36]–[40]. The design of our study allowed investigation of HCV infection according to sex and age; no statistically significant difference in HCV distribution was found according to sex, as for community-acquired hepatitis C in other regions of the world [41]–[43]. In our study, a history of parenteral injections, past hospital admission, and age >55 years were independent risk factors for HCV infection. Similar findings were reported in neighbouring Cameroon [10], Central African Republic [9], and the Republic of Congo [5]. These results confirm that the spread of HCV in central Africa is due to a cohort effect, with previous, possibly iatrogenic exposure. Iatrogenic events associated with massive and generalized therapy and/or vaccination have recently been shown to be a major cause of transmission of HCV in Egypt [3], Cameroon [7], [8], and the Central African Republic [9]. Such procedures were repeated annually, often with non-sterile equipment and serial arm-to-arm injections. The timing of these events is consistent with the pattern of age-dependent seroprevalence. More extensive epidemiological studies are needed to better characterize the route of transmission and dissemination of HCV in Gabon.

This is the first large study of HCV molecular epidemiology in Gabon. Our phylogenetic analysis indicates the circulation of three HCV genotypes (1, 2 and 4), with a predominance of genotype 4. In a previous study in Gabon, only HCV genotype 4 was found [6], [28]. The current study documents that there is high diversity in genotypes 1 and 4 and many unsubtyped sequences. We [14], [31] and others [44] have reported the circulation of three HCV genotypes (1, 2 and 4) in neighbouring Cameroon and Central African Republic, with greater genetic diversity within genotype 4. HCV genotype 4 also predominated and exhibited wide genetic diversity in previous studies in Gabon [6], nearby Cameroon [31], the Central African Republic [14] and the Republic of Congo [5]. An interesting finding is the difference in the predominant HCV-4 subtype in these four countries: subtype 4f in Cameroon [31], subtype 4e in Gabon ([6] and this study), subtype 4k in the Central African Republic [14] and 4c/4r in the Republic of Congo [5]. These five HCV-4 subtypes thus represent the signature HCV infection in the corresponding country. Characterization of the full genomes of these subtypes is needed for correct classification and further studies.

Phylogenetic concordance was found in all 59 strains in which both the NS5B and core regions were sequenced. Unlike in some previous reports [45]–[52], genotype/subtype recombinant sequences or dual genotype/subtype infection were not observed in this study, indicating that recombination is a rare event in HCV, as reported previously [53], [54].

The epidemic history of HCV-4e, -4f, -4c, -4t and -4r strains was studied with a coalescent approach to population genetics. The MRCA of subtype 4e in this study is older than that reported by Ndong-Atome et al. [6] in a remote village of Gabon. This suggests different epidemic histories of HCV-4e in Gabon, with different periods of introduction according to the administrative region. The MRCAs of subtypes 4f and 4c in this study are in the same range as that of Cameroon [15] and the Republic of Congo [5], suggesting a similar period of introduction. Interestingly, the MRCA of subtype 4k in this study is more recent than that reported by Njouom et al. [14] in a village in the Central African Republic, suggesting that this subtype was introduced into Gabon from that country. The similarities in the population histories of HCV-4 in Gabon, Cameroon, the Central African Republic, and the Republic of Congo, despite differences in the predominant subtype, suggest that medical interventions amplified the HCV-4 subtype, which was introduced into cohorts of patients during mass intravenous drug campaigns by mobile teams at health centers or in villages. Less common subtypes presumably correspond to those for which such amplification did not occur or which occurred only at the end of the era of massive administration of intravenous drugs, with fewer cycles of amplification.

In conclusion, this study shows that, like neighbouring Cameroon, the Central African Republic, and the Republic of the Congo, Gabon has a high prevalence of HCV, especially among older people, and, moreover, that it is highly heterogeneic (ranging from 3.7% to 20.8%, depending on the area studied). Fourteen subtypes, including nine subtype 4 strains, were identified, suggesting that genotype 4, especially subtype 4e, which predominates, has been endemic for a long time in Gabon. Coalescence studies of subtypes 4e, 4f, and 4c with an epidemic profile indicate that they spread exponentially during the first part of the 20th century, probably due to iatrogenic transmission, as reported in previous studies. In contrast, the 4t and 4k populations did not reach growth equilibrium during this period and continue to spread. Further studies are required to identity the risk factors for transmission in the country.
